# Survival predictors in anuric patients on peritoneal dialysis: A prospective, multicenter, propensity score-matched cohort study

**DOI:** 10.1371/journal.pone.0196294

**Published:** 2018-04-25

**Authors:** Ja-Yong Park, Jang-Hee Cho, Hye Min Jang, Yon Su Kim, Shin-Wook Kang, Chul Woo Yang, Nam-Ho Kim, Ji-Young Choi, Sun-Hee Park, Chan-Duck Kim, Yong-Lim Kim

**Affiliations:** 1 Clinical Research Center for End-Stage Renal Disease (CRC for ESRD) in Korea; 2 School of Medicine, Kyungpook National University, Daegu, Korea; 3 Seoul National University College of Medicine, Seoul, Korea; 4 Yonsei University College of Medicine, Seoul, Korea; 5 Catholic University of Korea College of Medicine, Seoul, Korea; 6 Chonnam National University Medical School, Gwangju, Korea; Postgraduate Medical Institute, INDIA

## Abstract

Prevalent anuric peritoneal dialysis (PD) patients usually have higher mortality than PD patients with residual urine volume. We aimed to evaluate the predictors of survival in anuric PD patients. Anuric PD patients (n = 505, <100 mL of daily urine) enrolled in Korean nationwide prospective cohort were analyzed. Survived and non-survived anuric PD patients were compared by propensity score matching analysis with a ratio of two to one. The propensity method was used to adjust for patient age, dialysis duration, and presence of diabetes. Among the total anuric PD patients, non-survived patients showed a significantly older age, higher incidence of diabetes, coronary artery disease, and arrhythmia, and lower serum creatinine and albumin. After propensity score matching, multivariate Cox regression analysis for patient survival showed a decreasing risk as serum albumin increased (HR = 0.347, p = 0.0094). Analysis using the receiver-operating-characteristic (ROC) curve showed that survival could be predicted with a sensitivity of 59.4% and a specificity of 63.2% using a cutoff value of 3.6 g/dL of serum albumin in unmatched total PD patients. The beneficial impact of high albumin level on death was significantly greater for patients with older age (≥50 years), no diabetes, low ultrafiltration (UF) volume (<1000 mL/day), and low levels of serum creatinine (<10 mg/dL), total cholesterol (<177.5 mg/dL), ferritin (<100 ng/mL), and high-sensitivity C-reactive protein (hs-CRP) (<0.1 mg/dL). Survival in anuric PD patients was associated with age, comorbidities, and nutritional factors such as creatinine and albumin. After adjustment by propensity score matching, serum albumin level was an independent predictor for survival in anuric PD patients.

## Introduction

Patients with end-stage renal disease (ESRD) show much lower survival rate compared to general population of the same age [1]. Peritoneal dialysis (PD) is an established treatment modality for patients with ESRD. In several retrospective and prospective cohort studies, predictors of outcome in patients treated with PD have been investigated. The survival of patients on PD is associated with various clinical factors such as demographic findings, comorbidities, nutritional markers, and peritoneal clearance [2–5].

For each 1-year increase in age, the risk of death increases by 4%, and patients with diabetes have a 30% increased risk of death compared with non-diabetic patients [6]. Cardiovascular disease accounts for most deaths in dialysis patients (approximately 50%). In addition, malnutrition is a negative predictor of survival in patients with ESRD. Fluid status and volume homeostasis are important in patients on PD, and a number of studies have shown that fluid overload is prevalent in patients on PD. Inadequate fluid removal in this population contributes to hypertension and is associated with an increased risk of cardiovascular disease, hypoalbuminemia, and systemic inflammation [7–9]. However, this effect was found to depend mainly on the contribution of residual glomerular filtration rate (GFR) [5]. Regardless of how long the residual renal function can be maintained in patients on PD, patients will ultimately lose their residual renal function completely.

Prevalent PD patients might have higher mortality rate when they become anuric during dialysis. The purpose of this study was to identify the factors that predict survival in anuric patients on PD.

## Materials and methods

### Patients and data collection

We conducted a prospective observational cohort study on Korean dialysis patients at the Clinical Research Center for End-Stage Renal Disease (CRC for ERSD, NCT00931970). Patients who were at least 20 years old and who began treatment with maintenance PD because of ESRD within 3 months were included for the study. Patients scheduled to receive kidney transplantation within 3 months were excluded. Between September 2008 and June 2011, 505 anuric patients on continuous ambulatory peritoneal dialysis (CAPD) were recruited into the study with the mean follow-up duration of 21.4±7.9 months. Anuria was defined as a 24-h urine output lower than 100 mL. Demographic variables at enrollment included age, gender, duration of dialysis, primary renal disease, comorbidities, laboratory data, and dialysis information. Comorbid conditions included chronic lung disease, coronary artery disease, peripheral vascular disease, cerebrovascular disease, diabetes mellitus, congestive heart failure, arrhythmia, connective tissue disease, peptic ulcer disease, mild liver disease, and moderate to severe chronic liver disease. Laboratory data were available for hemoglobin, serum blood urea nitrogen (BUN), creatinine, albumin, total cholesterol, triglyceride, ferritin, and high-sensitivity C-reactive protein (hs-CRP). PD information included membrane transport status, UF volume, weekly dialysate Kt/Vurea, body mass index (BMI), and blood pressure. The erythropoiesis-stimulating agents used in this study were epoetin-α and darbepoetin, using a conventional ratio of 200:1 to convert from epoetin-α to darbepoetin. Comorbidities, laboratory data, and dialysis information were followed at 3 and 6 months after the start of renal replacement therapy, and then at 6-month intervals thereafter. The present study used the data at the recruitment period of anuric PD patients. Patient’s death, a primary end point of the study, was reported within 1 month after the event and ascertained by data from Statistics Korea. The study had local ethical committee clearance at all sites. Written informed consent was obtained from all studied subjects before inclusion. The Institutional Review Board of Kyungpook National University Hospital approved the study protocol, and all clinical investigations were conducted in accordance with the guidelines of the 2008 Declaration of Helsinki.

### Statistical analysis

Results of continuous variables were expressed as mean ± standard deviation (SD) for normal distribution. For categorical variables, the results were expressed as frequencies and percentages. Comparisons of continuous variables and categorical data between the two study groups were tested by the *t*-test and Chi-square test.

To increase the power of the statistical analysis, we constructed a propensity score-matching model. The model included age, comorbid diabetes at baseline, and dialysis vintage. Propensity score matching was conducted using a 2:1 matching procedure, whereby each non-survived anuric patient on PD was matched with two survived anuric patients on PD. The predictors for survival were analyzed using a multivariate Cox’s proportional-hazard model in which all the significant variables from the univariate analysis were included. Proportional hazard assumptions were tested using Schoenfeld residuals. Survival curves were generated by the Kaplan-Meier method. For the comparison of two scales as predictors for hypoalbuminemia, a receiver operating characteristic (ROC) curves analysis was performed.

We performed subgroup analyses in patients categorized by age (<50 years, ≥50 years), gender, diabetes, dialysis vintage (<5 years, ≥5 years), UF volume (<1000 mL/day, ≥1000 mL/day), hemoglobin (<10 g/dL, ≥10 g/dL), serum creatinine (<10 mg/dL, ≥10 mg/dL), total cholesterol (<177.5 mg/dL, ≥177.5 mg/dL), ferritin (<100 mg/dL, ≥100 mg/dL), hs-CRP (<0.1 mg/dL, ≥0.1 mg/dL). All statistical analyses were performed using SAS system for Windows, version 9.2 (SAS Institute Inc., Cary, NC). Differences were regarded as statistically significant when *p* < 0.05.

## Results

Demographic data of the study population are shown in Table 1. Among the 505 patients on CAPD in the study, the mean age was 50.29±12.94 years, and the duration of PD was 71.50±50.34 months. Diabetes was the most common cause of ESRD (39.56%), followed by hypertension (28.40%), glomerulonephritis (25.24%), and others (6.80%). By the end of follow-up, 61 (12.07%) patients had died, and 444 (87.93%) patients were alive. Compared to non-survived patients, survived patients had a lower number of risk factors including older age (49.04±12.66 vs. 59.44±11.23, p<0.0001), diabetes as the cause of renal failure (35.65% vs. 66.04%, p<0.0001), and cardiovascular diseases such as atrial fibrillation and coronary artery disease (p<0.05, Table 1). In addition, survived patients had significantly higher serum creatinine (11.40±7.23 vs. 9.90±3.17, p = 0.0053) and serum albumin (3.75±1.62 vs. 3.44±0.45, p = 0.0016) levels than non-survived patients (Table 2).

**Table 1:** Demographics of anuric patients on PD.

	Total (N = 505)	Survived (N = 444)	Non-survived (N = 61)	P-value
Dialysis vintage (months)	71.50±50.34	71.17±50.96	73.85±46.02	0.6975
Age at entry (years)	50.29±12.94	49.04±12.66	59.44±11.23	<0.0001
Gender (% male)	289 (57.23)	255 (57.43)	34 (55.74)	0.8019
Primary renal disease (%)				<0.0001
Diabetes	163 (39.56)	128 (35.65)	35 (66.04)	
Hypertension	117 (28.40)	103 (28.69)	14 (26.42)	
Glomerulonephritis	104 (25.24)	101 (28.13)	3 (5.6)	
Others	28 (6.80)	27 (7.52)	1 (1.89)	
Co-morbidity				
Chronic lung disease	20 (5.78)	15 (4.97)	5 (11.36)	0.1550
Coronary artery disease	21 (6.07)	15 (4.97)	6 (13.64)	0.0371
Peripheral vascular disease	7 (2.03)	6 (1.99)	1 (2.33)	1.0000
Cerebrovascular disease	29 (8.38)	23 (7.62)	6 (13.64)	0.2371
Diabetes	189 (46.32)	151 (42.90)	38 (67.86)	0.0005
Congestive heart failure	28 (8.09)	21 (6.95)	7 (15.91)	0.0678
Arrhythmia	6 (1.73)	3 (0.99)	3 (6.82)	0.0292
Connective tissue disease	24 (6.94)	22 (7.28)	2 (4.55)	0.7522
Peptic ulcer disease	13 (3.76)	11 (3.64)	2 (4.55)	0.6746
Mild liver disease	18 (5.22)	13 (4.32)	5 (11.36)	0.0642
Moderate or severe liver disease	10 (2.89)	10 (3.31)	0 (0.00)	0.6220

**Table 2:** Biochemical variables of anuric patients on PD.

	Total (N = 505)	Survived (N = 444)	Non-survived (N = 61)	P-value
Membrane transport status				0.1235
High	41 (25.15)	30 (22.06)	11 (40.74)	
High average/low average	115 (70.55)	100 (73.53)	15 (55.56)	
Low	7 (4.29)	6 (4.41)	1 (3.70)	
UF volume (ml/day)	1112.07±699.24	1130.80±705.34	992.30±652.49	0.1803
Dialysate only weekly Kt/V	1.69±0.56	1.68±0.57	1.75±0.51	0.5425
BMI (kg/M^2^)	23.40±3.27	23.38±3.22	23.59±3.66	0.6514
Systolic BP (mmHg)	134.69±23.94	134.53±23.52	135.85±26.99	0.7006
Diastolic BP (mmHg)	82.13±35.12	82.73±37.06	77.82±14.22	0.0673
Hemoglobin (g/dL)	10.48±5.00	10.51±5.32	10.27±1.28	0.4325
BUN (mg/dL)	60.11±26.92	60.63±27.50	56.37±22.12	0.1758
Serum creatinine (mg/dL)	11.22±6.89	11.40±7.23	9.90±3.17	0.0053
Serum albumin (g/dL)	3.71±1.53	3.75±1.62	3.44±0.45	0.0016
Total cholesterol (mg/dL)	176.92±42.65	177.88±42.72	169.90±41.81	0.1815
Triglyceride (mg/dL)	155.49±107.76	157.38±111.48	142.63±77.64	0.2625
LVH on EKG				0.9497
Yes	70 (27.34)	60 (27.27)	10 (27.78)	
No	186 (72.66)	160 (72.73)	26 (72.22)	
Cardiomegaly on CXR				0.7837
Yes	80 (32.26)	69 (31.94)	11 (34.38)	
No	168 (67.74)	147 (68.06)	21 (65.63)	
Ejection fraction (%)	56.74±11.12	57.49±10.42	47.79±35.00	0.1436
Ferritin (ng/mL)	256.55±298.54	263.87±312.34	211.68±191.26	0.2096
ESA dose (U/week)	5352.3±2669.6	5439.9±2740.9	4809.7±2134.2	0.2234
hs-CRP (mg/dL)	0.89±2.22	0.89±2.31	0.88±1.49	0.9810

Abbreviations: UF, ultrafiltration; BMI, body mass index; BUN, blood urea nitrogen; LVH, left ventricular hypertrophy; CXR, chest X-ray; ESA, erythropoiesis-stimulating agent.

Univariate Cox regression analysis showed an increasing hazard ratio (HR) for patient survival with increasing patient age, decreasing duration of dialysis, and the presence of accompanying coronary artery disease or diabetes. Additionally, as UF volume, serum creatinine, and serum albumin increased, the HR decreased. Multivariate analysis conducted for these data showed increased HR with significant differences according to patient age (HR = 1.049, p = 0.0034) and diabetes (HR = 2.144, p = 0.0421), and with a decreased HR according to dialysis vintage (HR = 0.976, p = 0.0393) and serum albumin (HR = 0.471, p = 0.0480) (Table 3).

**Table 3:** Univariate and multivariate Cox regression analyses of patient survival in anuric patients on PD.

	Univariate analysis		Multivariate analysis	
	HR (95% CI)	P-value	HR (95% CI)	P-value
Age at entry (years)	1.101 (1.076–1.128)	<0.0001	1.049 (1.016–1.083)	0.0034
Dialysis vintage (months)	0.948 (0.936–0.959)	<0.0001	0.976 (0.954–0.999)	0.0393
Gender (female vs. male)	0.829 (0.499–1.378)	0.4693		
Coronary artery disease	4.282 (1.791–10.241)	0.0011	1.245 (0.436–3.558)	0.6819
Diabetes	4.758 (2.627–8.618)	<0.0001	2.144 (1.027–4.472)	0.0421
BMI (kg/M^2^)	1.076 (0.997–1.162)	0.0602		
Systolic BP (mmHg)	1.009 (0.997–1.021)	0.1558		
Diastolic BP (mmHg)	0.992 (0.972–1.013)	0.4459		
Hemoglobin (g/dL)	0.974 (0.882–1.074)	0.5960		
BUN (mg/dL)	0.996 (0.984–1.008)	0.5312		
Serum creatinine (mg/dL)	0.845 (0.779–0.917)	<0.0001	0.97 (0.869–1.082)	0.5801
UF volume (L/day)	0.557 (0.358–0.865)	0.0093	0.793 (0.487–1.29)	0.3500
Serum albumin (g/dL)	0.387 (0.261–0.573)	<0.0001	0.473 (0.224–1.002)	0.0505
Total cholesterol (mg/dL)	0.990 (0.989–1.002)	0.1496		
Ferritin (ng/mL)	1.000 (0.999–1.001)	0.9341		
hs-CRP (mg/dL)	1.015 (0.845–1.219)	0.8712		
Membrane transport status				
Slow average / Slow	Reference			
Fast / Fast average	2.270(0.854–6.033)	0.1002		

Propensity score analysis was conducted with ratio of 2:1 of survived patients to non-survived patients. Dialysis vintage, age at entry, and diabetes were used as variables for adjustment in the propensity score matching. The analysis was performed in 61 non-survived patients and 122 propensity score-matched survived patients (Table 4). After propensity score matching, survived patients showed higher serum albumin (3.63±0.46 vs. 3.44±0.45, p = 0.0111) and total cholesterol (184.79±45.83 vs. 169.90±41.81, p = 0.0390) levels with significant differences. However, other variables did not show any significant differences between the two groups (Table 5).

**Table 4:** Demographics of anuric patients on PD (matched cohort).

Variables	Total (N = 183)	Survived (N = 122)	Non-survived (N = 61)	P-value
Dialysis vintage (months)	67.06±43.66	63.67±42.21	73.85±46.02	0.1375
Age at entry (years)	57.66±13.28	56.78±14.16	59.44±11.23	0.1692
Gender (% male)	106 (57.92)	72 (59.02)	34 (55.74)	0.6719
Primary renal disease (%)				0.1117
Diabetes	95 (62.50)	60 (60.61)	35 (66.04)	
Hypertension	31 (20.39)	17 (17.17)	14 (26.42)	
Glomerulonephritis	18 (11.84)	15 (15.15)	3 (5.66)	
Others	8 (5.26)	7 (7.07)	1 (1.89)	
Co-morbidity				
Chronic lung disease	13 (9.56)	8 (8.70)	5 (11.36)	0.7562
Coronary artery disease	14 (10.29)	8 (8.70)	6 (13.64)	0.3802
Peripheral vascular disease	3 (2.22)	2 (2.17)	1 (2.33)	1.0000
Cerebro-vascular disease	15 (11.03)	9 (9.78)	6 (13.64)	0.5624
Diabetes	115 (67.25)	77 (66.96)	38 (67.86)	0.9062
Congestive heart failure	16 (11.76)	9 (9.78)	7 (15.91)	0.2995
Arrhythmia	4 (2.94)	3 (6.82)	1 (1.09)	0.0993
Connective tissue disease	6 (4.41)	4 (4.35)	2 (4.55)	1.0000
Peptic ulcer disease	6 (4.41)	4 (4.35)	2 (4.55)	1.0000
Mild liver disease	10 (7.35)	5 (5.46)	5 (11.36)	0.2922
Moderate or severe liver disease	3(2.21)	3 (3.26)	0 (0.00)	0.5508

**Table 5:** Biochemical variables of anuric patients on PD (matched cohort).

Variables	Total (N = 183)	Survived (N = 122)	Non-survived (N = 61)	P-value
Membrane transport status				0.3847
High	19 (31.67)	8 (24.24)	11 (40.74)	
High average/low average	38 (63.33)	23 (69.70)	15 (55.56)	
Low	3 (5.00)	2 (6.06)	1 (3.70)	
UF volume (ml/day)	1022.06 ±708.89	1039.98 ±743.87	992.30 ±652.49	0.7004
Dialysate only weekly Kt/V	1.66±0.52	1.58±0.53	1.75±0.51	0.1891
BMI (kg/M^2^)	23.73±3.42	23.80±3.30	23.59±3.66	0.6984
Systolic BP (mmHg)	134.38±25.47	133.63±24.74	135.85±26.99	0.5995
Diastolic BP (mmHg)	83.21±56.28	85.98±68.44	77.82±14.22	0.2383
Hemoglobin (g/dL)	10.17±1.47	10.13±1.57	10.27±1.28	0.5309
BUN (mg/dL)	56.12±19.68	56.00±18.43	56.37±22.12	0.9056
Serum creatinine (mg/dL)	9.77±3.39	9.70±3.51	9.90±3.17	0.7145
Serum albumin (g/dL)	3.56±0.46	3.63±0.46	3.44±0.45	0.0111
Total cholesterol (mg/dL)	179.83±44.96	184.79±45.83	169.90±41.81	0.0390
Triglyceride (mg/dL)	159.26±102.25	168.37±112.88	142.63±77.64	0.1285
LVH on EKG				0.9502
Yes	29 (28.16)	19 (28.36)	10 (27.78)	
No	74 (71.84)	48 (71.64)	26 (72.22)	
Cardiomegaly on CXR				0.4165
Yes	29 (29.00)	18 (26.47)	11 (34.38)	
No	71 (71.00)	50 (73.53)	21 (65.63)	
Ejection fraction (%)	52.94±11.09	54.15±9.62	47.67±17.79	0.3793
Ferritin (ng/mL)	271.12±360.31	304.62±425.27	211.68±191.26	0.1682
ESA dose (U/week)	5583.1±2582.8	5718.5±2776.6	4809.7±2134.2	0.1203
hs-CRP (mg/dL)	1.16±2.68	1.28±3.06	0.88±1.49	0.4384

In addition, the univariate Cox regression analysis for patient survival showed a decreasing risk with increasing dialysis vintage (HR = 0.982, p = 0.0477), serum albumin (HR = 0.444, p = 0.0083) and total cholesterol (HR = 0.993, p = 0.0371), while no significant differences were observed for the other variables. Multivariate analysis showed a decreasing risk with increasing dialysis vintage (HR = 0.906, p<0.0001) and serum albumin (HR = 0.347, p = 0.0094), though total cholesterol level showed no significant difference between the two groups (HR = 0.992, p = 0.0600) (Table 6).

**Table 6:** Relative risk of death in anuric patients on PD (matched cohort).

	Univariate analysis		Multivariate analysis	
Variables	HR (95% CI)	P-value	HR (95% CI)	P-value
Age at entry (years)	1.022 (0.998–1.047)	0.0724	1.000 (0.975–1.026)	0.986
Dialysis vintage (months)	0.982 (0.964–1.000)	0.0477	0.906 (0.877–0.936)	<0.0001
Gender (female vs. male)	0.888 (0.524–1.505)	0.6593		
Coronary artery disease	1.909 (0.771–4.728)	0.1623		
Diabetes	1.335 (0.722–2.470)	0.3574		
BMI (kg/M^2^)	1.028 (0.946–1.116)	0.5169		
Systolic BP (mmHg)	1.007 (0.996–1.018)	0.2231		
Diastolic BP (mmHg)	0.997 (0.987–1.007)	0.5142		
Hemoglobin (g/dL)	1.050 (0.863–1.277)	0.6249		
BUN (mg/dL)	1.006 (0.993–1.020)	0.3633		
Serum creatinine (mg/dL)	1.004 (0.924–1.090)	0.9315		
UF volume (L/day)	0.898 (0.605–1.334)	0.5942		
Serum albumin (g/dL)	0.444 (0.243–0.811)	0.0083	0.347 (0.156–0.771)	0.0094
Total cholesterol (mg/dL)	0.993 (0.986–1.000)	0.0371	0.992 (0.984–1.000)	0.0600
Ferritin (ng/mL)	1.000 (0.998–1.001)	0.4613		
hs-CRP (mg/dL)	0.45 (0.755–1.181)	0.6175		
Membrane transport status				
High	Reference			
High average / low average	1.020 (0.446–2.332)	0.9630		
Low				

**Fig 1:**
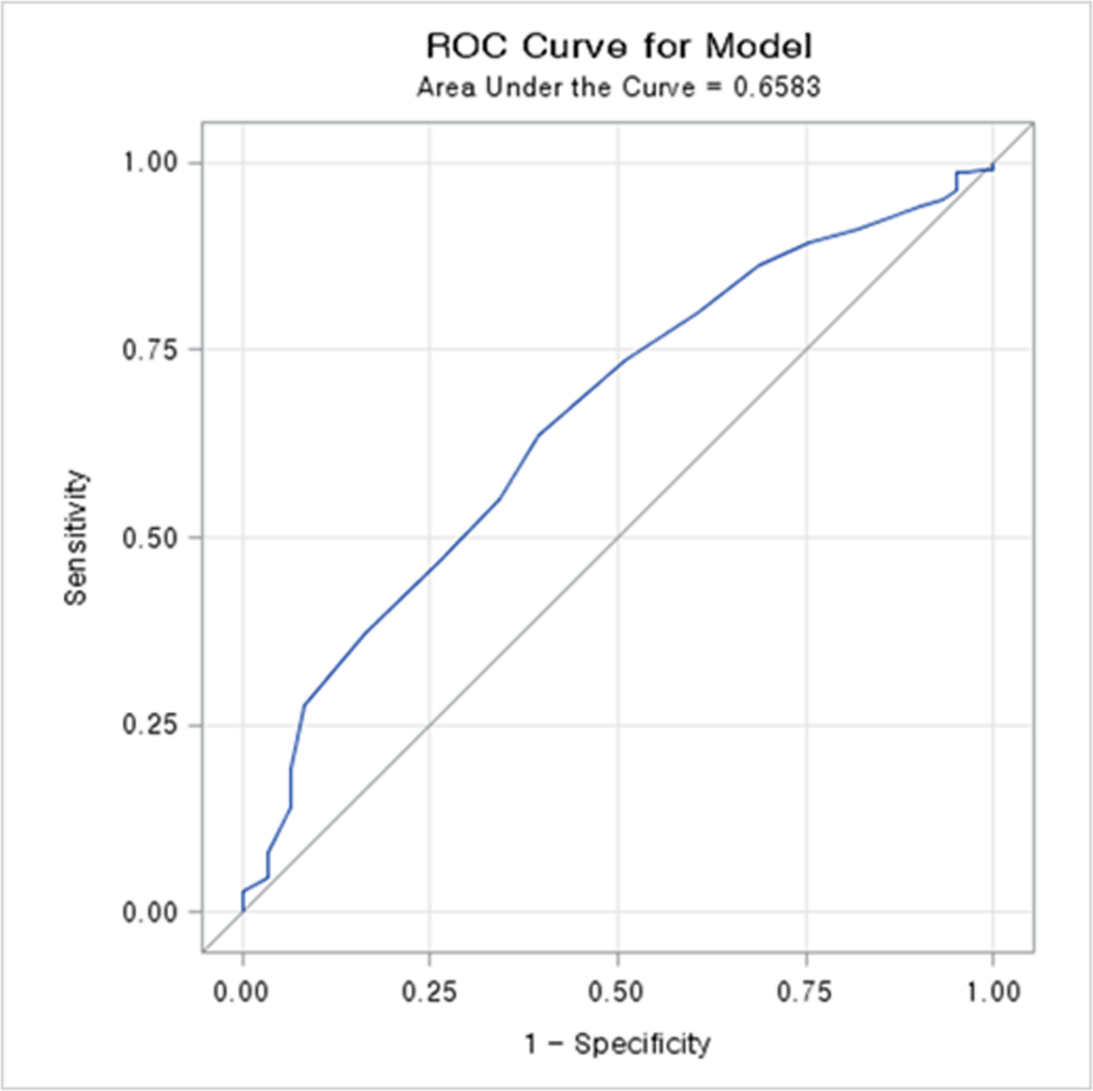
The receiver operating characteristic (ROC) curve for the prediction of death according to serum albumin level. Serum albumin levels below 3.6 g/dL predicted adverse renal outcome with a 59.4% sensitivity and a 63.2% specificity.

**Fig 2:**
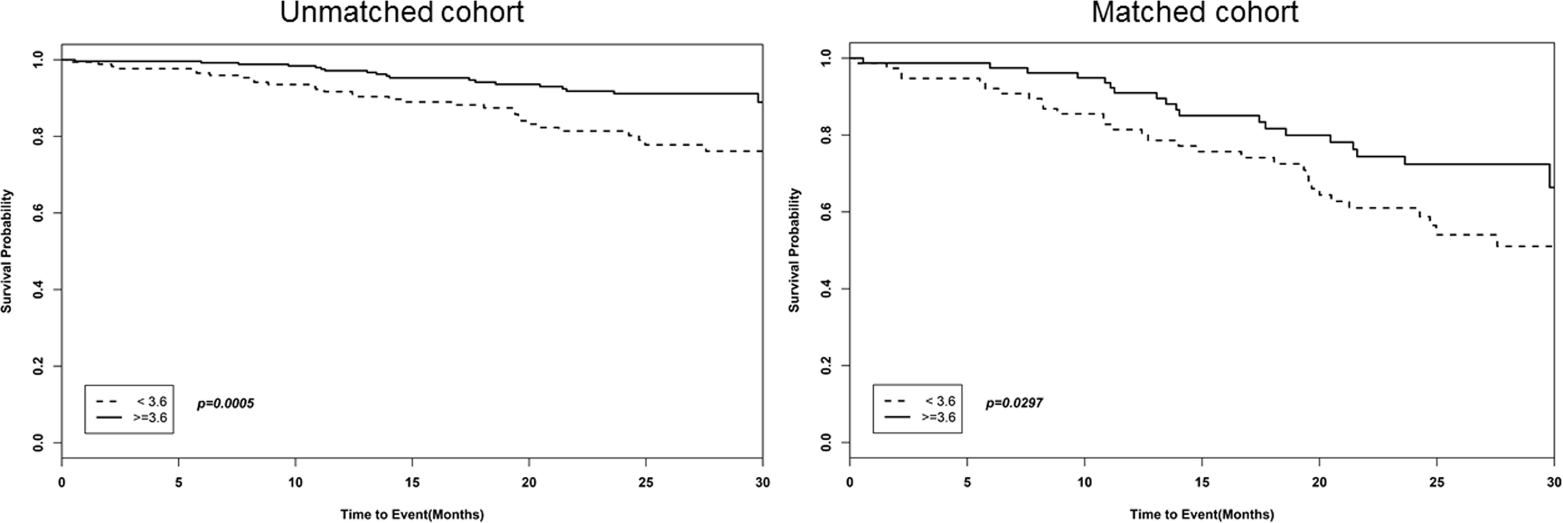
Kaplan-Meier survival curve according to serum albumin level in the unmatched and matched cohorts.

Analysis using the ROC curve for predicting death according to serum albumin level showed that death could be predicted with a sensitivity of 59.4% and a specificity of 63.2% using a cutoff serum albumin value of 3.6 g/dL. The areas under the curves of serum albumin for predicting survival was 0.658, demonstrating that serum albumin level could be considered as a fair predictor of survival. (Fig 1). When the serum albumin level of 3.6 g/dL was set as the standard, significant differences in the survival rate were observed in unmatched and matched groups (p = 0.0005 and p = 00297) (Fig 2).

**Fig 3:**
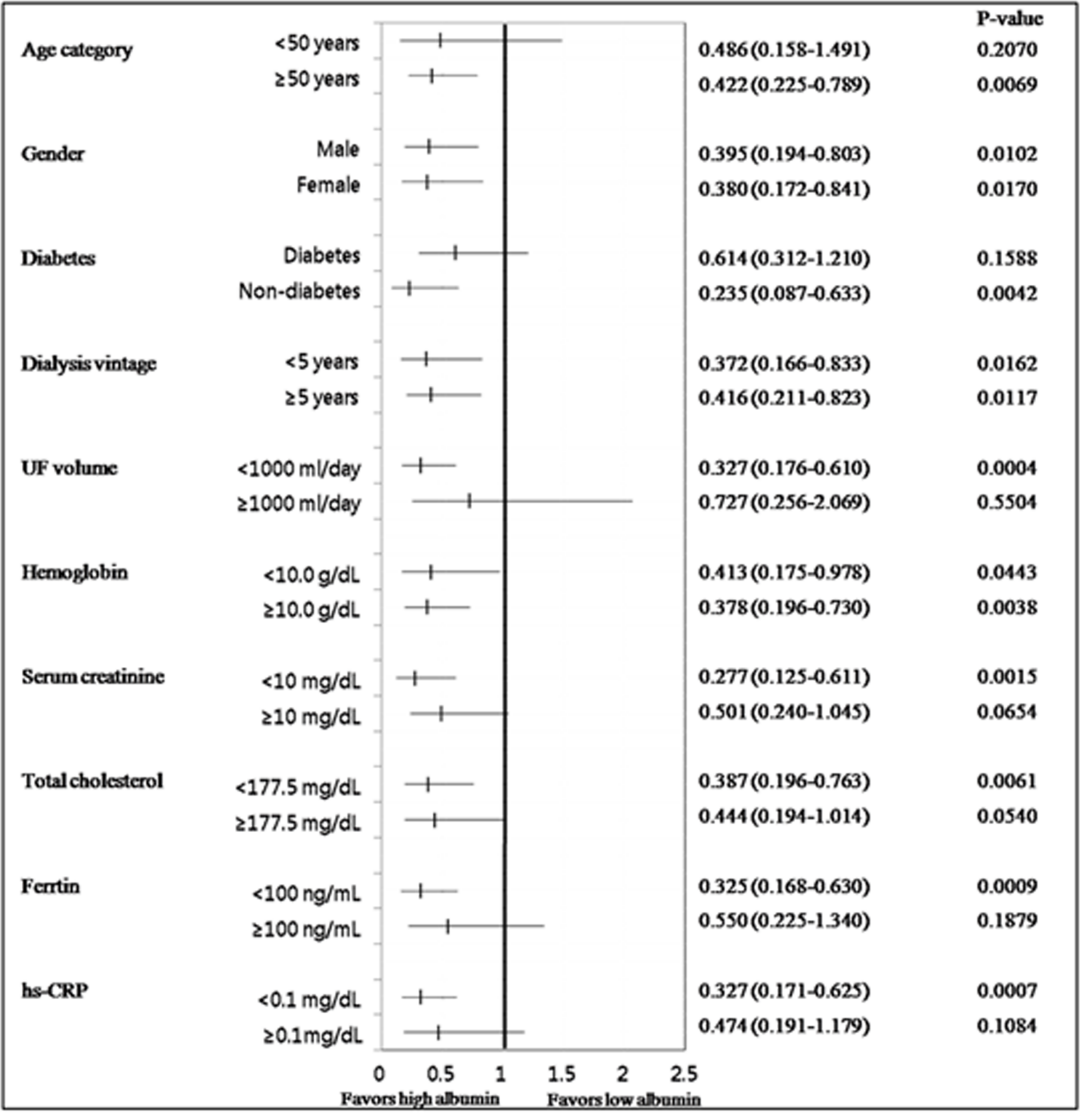
Forest plot showing the adjusted hazard ratios for death in the unmatched cohort.

The positive association between high albumin level and survival was significantly greater for patients with older age (≥50 years), no diabetes, low UF volume (<1000 mL/day), and low levels of serum creatinine (<10 mg/dL), total cholesterol (<177.5 mg/dL), ferritin (<100 ng/mL), and hs-CRP (<0.1 mg/dL). In the context of albumin levels greater than 3.6 g/dL, a significantly increased survival rate was observed regardless of the sex, duration of dialysis, or hemoglobin level of the patients (Fig 3).

## Discussion

In this study, based on the results of multicenter prospective observation studies on dialysis patients in Korea, we implemented analyses of the factors that might influence prognosis in anuric patients on PD. Using propensity score matching, analyses with a ratio of 2:1 of survived patients to non-survived patients were conducted according to the differences in patient age, duration of dialysis, and the presence of diabetes. Serum albumin level was found to be an independent predictor for survival in anuric patients on PD. This is the largest prospective study ever published which investigated survival predictors in anuric PD patients.

The increasing dialysis vintage is associated with declining body weight and deterioration of nutritional status, which may affect survival of patients on PD [10]. Because the present study included only the anuric PD patients at the recruitment, the exact duration of dialysis until anuria could not be evaluated. It is difficult to draw a definite conclusion from the univariate and multivariate analysis with duration of dialysis. Nevertheless, the duration of dialysis could be correlated with duration on dialysis of patients when they became anuric, especially when the duration is short. Therefore, relationship between dialysis duration and survival shown in our study might suggest that faster the patient becomes anuric, more likely the patient would not survive long. This has been reported in many studies suggesting preserving residual renal function is an important factor for longer survival in PD patients [11–13].

Nutritional markers are known to be as an important factor for survival in patients on PD [14–16], and among them, serum albumin concentration is often used as a surrogate measure of malnutrition. However, the utility of albumin as a marker of malnutrition in PD has remained controversial. The association between other measures of nutrition and albumin concentration is inconsistent, which may be due to multiple factors. Albumin is a negative acute-phase reactant and its production is suppressed in inflammatory states [17]. In addition to inflammation, peritoneal albumin loss and volume overloading can lead to hypoalbuminemia in patients on PD [18]. Although serum albumin concentration is not an effective isolated marker of nutritional status because of its multiple confounding factors, our study showed that serum albumin level may be considered an important and meaningful factor to facilitate the prediction of prognosis in anuric patients on PD. Jansen et al. also reported that several risk factors such as low serum albumin as well as old age, comorbidity, and dialysis vintage, were significantly associated with worse patient survival [19]. However, they studied with relatively small number of anuric patients, to which comparison with propensity matching could not be applied. Moreover, we found that serum albumin concentration may differently affect patient survival according to age, status of diabetes, UF volume, serum creatinine level, total cholesterol level, ferritin level, and hs-CRP level. To ascertain the cause of the varying effects of hypoalbuminemia, further studies that elucidate the relationship between albumin and these factors are warranted.

Increased serum creatinine level has been associated with good survival, whereas lower serum creatinine level has been associated with increased mortality in hemodialysis patients [20, 21]. These findings suggest that serum creatinine level reflects muscle mass and that low muscle mass resulting from malnutrition is associated with poor outcomes. In a previous study, the association between serum creatinine level and survival was observed in a retrospective PD cohort [22]. However, that previous study has a limitation in that residual renal function, which can directly affect serum creatinine level, was unable to be examined. Residual renal function may be better preserved and may contribute more significantly to total creatinine clearance, particularly in patients with a shorter PD duration [23, 24]. Given that residual GFR is an important predictor of survival, the association between serum creatinine and survival may be modified by residual GFR. Since our present study targeted anuric patients with PD, it was possible to exclude the influence of residual renal function on serum creatinine. In the unmatched cohort analysis, survived patients showed increased serum creatinine and a statistically significant difference in the survival rate, which increased with increasing serum creatinine levels and was confirmed in the Kaplan-Meier survival curves. These findings are in accordance with the results of previous studies. However, no significant difference in serum creatinine level was observed between the two groups in the propensity score analysis. This implies that serum creatinine may be used as a marker of nutritional status in patients with ESRD, but that it is not appropriate for predicting survival, especially anuric PD patients. Further studies are needed to verify the association between survival rate and serum creatinine level in anuric patients on PD.

The optimal UF volume of anuric patients on PD remains unknown [25]. The European Best Practice Guideline Working Group on PD recommends the minimum net UF of 1 L/day in anuric patients on PD [26]. However, the International Society for Peritoneal Dialysis states that no definite target for UF can be suggested from the previous studies [27]. In the same context, our study revealed no significant difference in survival rate according to the UF volume was observed in the propensity score analysis. While current guidelines do not adequately address the optimal UF target for anuric patients on PD, optimizing therapy to meet UF goals remains an important clinical target.

Bhaskaran et al. performed a retrospective analysis of anuric peritoneal dialysis patients in Canada and were unable to find a significant effect of Kt/Vurea on the relative risk of death [28]. However, multivariate analysis of a prospective cohort study of anuric patients in Hong Kong showed a significant effect of Kt/Vurea on survival advantage [29]. Maarten et al. showed that peritoneal UF rate, Kt/Vurea <1.5/week, and creatinine clearance <40 L/week/1.73 m^2^ were associated with an increase in the relative risk of death. Furthermore, they suggested that a Kt/Vurea of 1.7/week and a creatinine clearance of at least 45 L/week were reasonable targets, which is in line with the results of the ADEMEX study in which a further increase in these solute transport levels was not found to lead to better patient survival. The Kidney Disease Outcomes Quality Initiative (KDOQI) provides specific guidelines for PD adequacy since higher small solute clearance is associated with improved survival on PD. According to our results, variations in peritoneal small solute clearances did not correspond to improved survival. Further studies that assess factors other than small-solute clearances are necessary to determine their effects on survival.

Numerous investigations have recently monitored certain biomarkers such as cardiac troponins and hs-CRP in addition to clinical data such as diabetes and hypertension in order to more clearly identify patients with coronary artery disease and enable the risk stratification of patients with ESRD [30, 31]. Several studies have shown that the elevated hs-CRP is related with cardiovascular disease and patient’s death [32–34], whereas other studies have reported no association between hs-CRP and survival [35, 36]. In the present study, significant differences in the prognosis of patients between the two groups according to blood pressure, presence of cardiomegaly in LV hypertrophy on EKG, and CXR were not observed, and similar results were shown in the propensity score analysis. In addition, in terms of hs-CRP level, there was no significant difference between the two groups, and a similar pattern was shown in the propensity score analysis. Previous study demonstrated the relationship between diminished residual renal function and increased level of CRP. However, there are several studies which failed to prove the correlation [37]. Therefore, the question of whether hs-CRP is predictive of clinical outcomes in patients on PD irrespective of peritoneal clearance and residual renal function remains controversial. Further research is necessary to confirm the relevance of hs-CRP level in the prognosis of anuric patients on PD. However, this study showed that low hs-CRP levels decreased the hazard ratio for survival in anuric patients on PD, with higher serum albumin levels.

In conclusion, our study showed that in the absence of residual renal function, serum albumin level is an independent factor that influences the survival of patients on PD. Additionally, patients with relatively older age, no diabetes, peritoneal UF of less than 1000 mL/day, and low serum creatinine, total cholesterol, serum ferritin, and hs-CRP levels, survival rate was found to increase along with increasing serum albumin level. The statistical significance of the results was fortified through propensity score matching that was conducted using prognosis factors gleaned from previous studies of patients on PD. However, further large-scale research is necessary to confirm the relevance of cardiovascular factors, nutritional prognostic factors, and peritoneal clearance in predicting the prognosis of anuric patients on PD.
